# Validity and reliability of balance assessment software using the Nintendo Wii balance board: usability and validation

**DOI:** 10.1186/1743-0003-11-99

**Published:** 2014-06-10

**Authors:** Dae-Sung Park, GyuChang Lee

**Affiliations:** 1 Department of Physical Therapy, Konyang University, Gwanjeodong-ro, Daejeon, Seo-gu, Republic of Korea; 2 Department of Physical Therapy, Kyungnam University, 7 kyungnamdaehak-ro, Masanhappo-gu, Changwon-si, Gyeongsangnam-do, Republic of Korea

**Keywords:** Balance, Centre of pressure, Force platform

## Abstract

**Background:**

A balance test provides important information such as the standard to judge an individual’s functional recovery or make the prediction of falls. The development of a tool for a balance test that is inexpensive and widely available is needed, especially in clinical settings. The Wii Balance Board (WBB) is designed to test balance, but there is little software used in balance tests, and there are few studies on reliability and validity. Thus, we developed a balance assessment software using the Nintendo Wii Balance Board, investigated its reliability and validity, and compared it with a laboratory-grade force platform.

**Methods:**

Twenty healthy adults participated in our study. The participants participated in the test for inter-rater reliability, intra-rater reliability, and concurrent validity. The tests were performed with balance assessment software using the Nintendo Wii balance board and a laboratory-grade force platform. Data such as Center of Pressure (COP) path length and COP velocity were acquired from the assessment systems. The inter-rater reliability, the intra-rater reliability, and concurrent validity were analyzed by an intraclass correlation coefficient (ICC) value and a standard error of measurement (SEM).

**Results:**

The inter-rater reliability (ICC: 0.89-0.79, SEM in path length: 7.14-1.90, SEM in velocity: 0.74-0.07), intra-rater reliability (ICC: 0.92-0.70, SEM in path length: 7.59-2.04, SEM in velocity: 0.80-0.07), and concurrent validity (ICC: 0.87-0.73, SEM in path length: 5.94-0.32, SEM in velocity: 0.62-0.08) were high in terms of COP path length and COP velocity.

**Conclusion:**

The balance assessment software incorporating the Nintendo Wii balance board was used in our study and was found to be a reliable assessment device. In clinical settings, the device can be remarkably inexpensive, portable, and convenient for the balance assessment.

## Background

Balance can be defined as the ability to maintain a stable posture for maximum time with minimal body sway, or the ability to maintain the body’s center of gravity over its base of support [1]. Impaired balance, particularly in the standing posture, may limit the activities in daily life [2]. A balance test can provide information that is important for the establishment of standards to judge an individual’s functional recovery and make prediction of falls [3]. Several assessment tools have been designed to test balance. There are a number of tools such as the Timed get Up and Go test [4], the Tinetti Performance Oriented Mobility Assessment [5], and the Berg Balance Scale [6]. These tools are regarded as objective assessment methods for obtaining reliable data, even without the use of any specific equipment [3,7]. However, some limitations are encountered in using these tools. One such limitation is the ceiling effect, in which variance in an independent variable is either not estimated or is estimated above a certain level, and so there is insufficient accuracy in detecting minute changes [3]. Therefore, some researchers have emphasized the necessity of designing new assessment tools to overcome these limitations [8,9].

Balance is generally tested quantitatively in laboratory experiments [10]. The most commonly used device is a force platform. When using this device, data about body sway, a factor related to balance, are obtained by recording the vertical force applied by the body on the force platform [7]. Tests using a force platform are generally performed in a static state [10]. Values tested using a force platform can explain body sway, through various variables [7]. However, force platform is time-consuming in terms of performance of tests and careful installation of related software [3]. Moreover, it is placed beneath the floor, which is not easily or conveniently transported, and it increases costs considerably [3]. Although a force platform is suitable for use in laboratory experiments, it is unsuitable for assessment of patients in clinical setting [3,7]. The development of a tool that is both user-friendly and inexpensive is needed for facilitating simple and efficient balance assessment in clinical settings.

Recently, the Wii, designed and marketed by Nintendo, has attracted considerable attention as a new-generation device. The Wii is a relatively new video game that demands complete body interaction from its players [11]. Thus, its use can improve health and well-being [12]. The Wii games involve challenging tasks for improving the activity level of users through stretching, muscle strengthening, aerobic exercise, and balance training [13]. Visual feedback is continuously provided by the system, and the completion of the tasks indicates the user’s overall performance [14]. The Wii has also been used to provide amusement and motivation to users, and it has been useful (it could be highly useful) in the development of a rehabilitation device for patients [13,15,16]. As an accessory of the Wii, the Wii Balance Board (WBB) is designed to test balance [15]. As the WBB includes four load cells that relay the coordinates of the user’s position in the form of the center of pressure (COP), a similarity can be observed between the WBB and force platforms [17]. The values of COP obtained from the WBB resemble those obtained from typical force platform [17]. Clark et al. [3] reported that the WBB showed high reliability and validity in balance assessment. In their study, they found that it was possible for the WBB to test balance by using LapVIEW to make the software. However, it may be difficult to commercialize it as an easily used assessment tool because it was not able to provide, even in this clinical setting. The Balancia software has been made useable even in the clinical setting by using both visual studio and C# which can be used easily. In order to test balance, the WBB can be useful in collecting and analyzing data, as in the case of similar laboratory level device such as the force platform. Moreover, the advantages of its use in laboratories or clinical settings over laboratory level force platform include lower cost and convenience. In order to implement WBB-based systems in clinical settings, a tool is required for testing balance using the WBB, and its reliability and validity need to be ensured.

Thus, we aim to investigate the reliability and validity of the WBB-based system by comparing the COP values obtained from software connected to the WBB with those (values) derived from a standard laboratory grade force platform. By using this method, this study attempted to investigate the possibility of using a WBB system as a tool of testing balance in clinical settings.

## Methods

### Participants

Twenty healthy adults, age 18 to 40 years, participated in the study (gender = 12 male, 8 female; age = 29.50 ± 4.38 years; height = 170.55 ± 5.98 cm; weight = 64.50 ± 10.03 kg). None of the participants had injuries or diseases of the musculoskeletal or nervous system or had been taking medications that would affect standing balance six months prior to participation. Participants provided informed consent, and all procedures were approved by National Rehabilitation Center Institutional Review Board.

### Data collection

**Figure 1:**
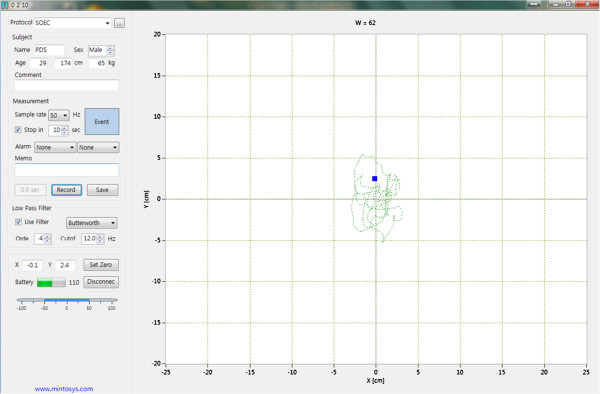
**Balancia software user interface.** A user can connect the WBB and Balancia software in a laptop equipped with Bluetooth for signal acquisition and analysis. Data were exchanged between the WBB and the laptop using the built-in Bluetooth and Balancia software.

The data collection was performed to recruit twenty healthy adults. The balance of participants was measured using the WBB-based system and laboratory grade force platform. The subjects and two assessors participated in the experiment for three days.The WBB-based system included the WBB, a laptop equipped with Bluetooth, and software (Balancia v1.0, Minto systems, Seoul, Republic of Korea) for signal acquisition and analysis, respectively. The size of the WBB equipped with four load cells was 45 × 26.5 cm. Data was exchanged between the WBB and the laptop using the built-in Bluetooth and Balancia software. Balancia software was developed using C++ and LabVIEW. The data was sampled at 50 Hz and filtered by a 4th order Butterworth low pass filter with a cut-off frequency of 12 Hz. The user interface of Balancia v1.0 is shown in Figure 1. The laboratory grade force platform (AMTI, Waterton, MA, USA) included six-axis load cells of four and had a size of 50 × 50 cm. The sampling rate was set at 50 Hz, and 12 Hz low-pass filtering was performed.

On the first day of the experiment, the participants participated in the 1^st^ day measurements with two assessors. Before the measurements, the general characteristics of the participants were collected through short interviews. Then, the first assessor measured the balance of the 20 participants using the WBB based system (A1), and the second assessor measured the balance of the same participants using WBB based system after providing them with sufficient rest (B). On the next day, the balance of the same participants was also measured by the first assessor using the WBB based system (A2). Finally, on the last day, the balance of the same participants was measured by the first assessor using the laboratory based force platform after 1 day of sufficient rest time (A3).

To investigate inter-rater reliability, the data with which the first and second assessor collected the balance of the same participants using the WBB based system was analyzed (A1 and B). The collections were performed by 2 experienced physical therapists as assessors. The assessors received instructions on the WBB-based system and laboratory grade force platform through guidelines made by developer at Balancia Software prior to the study. Each assessor instructed the participants in how to perform the maneuvers, guarded them, and measured each their performances before balance measurement. The instruction provided by each assessor was made to be consistent by the first assessor prior to the study, and the second assessor also received instructions from the first assessor. However, the two assessors did not speak to each other while doing measurements. Every effort was made to keep all factors like the area in which the test was conducted and the participant’s clothing consistent.

For intra-rater reliability, the data with which the first assessor repeatedly measured the balance of the same participants using the WBB based system was analyzed (A1 and A2). The collections were performed by the experienced physical therapist as first assessor. The assessor received instructions on the WBB-based system and laboratory grade force platform through guidelines made by developer at Balancia Software prior to the study to minimize a measurement error. The assessor also instructed the participants on how to perform the maneuvers, guarded them, and measured their performance on the first day as well as the second day. The instruction provided by the assessor was made to be consistent with those provided on the first day. In addition, every effort was made to keep all factors like the area in which test was conducted and the clothing consistent.

To investigate the concurrent validity, the data with which the first assessor repeatedly measured the balance of the participants using both the WBB based system and the laboratory based force platform was analyzed (A1 and A3). The collections were performed by the experienced physical therapists as first assessors too. The assessor received instructions on the WBB-based system and laboratory grade force platform through guidelines made by developer of Balancia Software prior to the study to minimize a measurement error. The assessor also instructed the participants on how to perform the maneuvers, guarded them, and measured each their performance before measurement on the first day as well as the third day. The instruction provided by the assessor was made to be consistent with those provided on the first day. In addition, every effort was made to keep all factors like the area in which test was conducted and the participant’s clothing consistent.

All measurements were performed according to four tasks that involved (1) standing on two legs with open eyes (STOE), (2) standing on two legs with closed eyes (STCE), (3) standing on one leg with open eyes (SOOE), and (4) standing on one leg with closed eyes (SOCE). Each task was performed thrice trials, with the standing on two legs performed for 30 s (sec), and the standing on one leg for 10 seconds (sec). A 10-sec break was given to the participants after each trial, and a minimum break of 60 sec was assumed for changing the task. For the tasks involving the standing on one leg, the participants were allowed to stand according to their dominant side, and for the tasks involving standing on two legs, the participants were allowed to maintain comfortable distance between their legs. The participants were asked to stand with arms folded, keep their hands placed on their chest and look straight ahead. If the participant appeared unstable even after this break, an additional break of a sufficient duration was provided before performing the next trial. The values of outcome measures were COP path length (PL) and COP velocity average (VA), and the mean of three repetitions were used.

### Statistical analysis

The Statistical Analysis SPSS v 17.0 (SPSS Inc. Chicago, IL, USA) was used. The inter-rater reliability, the intra-rater reliability, and concurrent validity were analyzed by intraclass correlation coefficient (ICC) values. The ICC values between 0.80 and 1.00 indicate high reliability, those between 0.60 and 0.79 indicate moderate reliability, and those below 0.60 indicate low reliability. Standard error of measurement was calculated to provide an estimate of the error in the units of measurement, thus giving clinically relevant values for expected error in an individual. The following equation was utilized to calculate the SEM; SEM = SD * (square root of (1-ICC)) [18]. In this equation, SD is the standard deviation of the measurement, and ICC is the reliability coefficient. For repeated measures, the SEM was multiplied by the square root of the number of measurements. SEM was also performed to examine inter-rater agreement by creating a Bland–Altman plot for the COP path lengths and average COP velocity of each measurement protocol [19]. It is used to graphically display the variability and systematic bias between two measurement sets; the 95% limits of agreement (LOAs).

## Results

**Figure 2:**
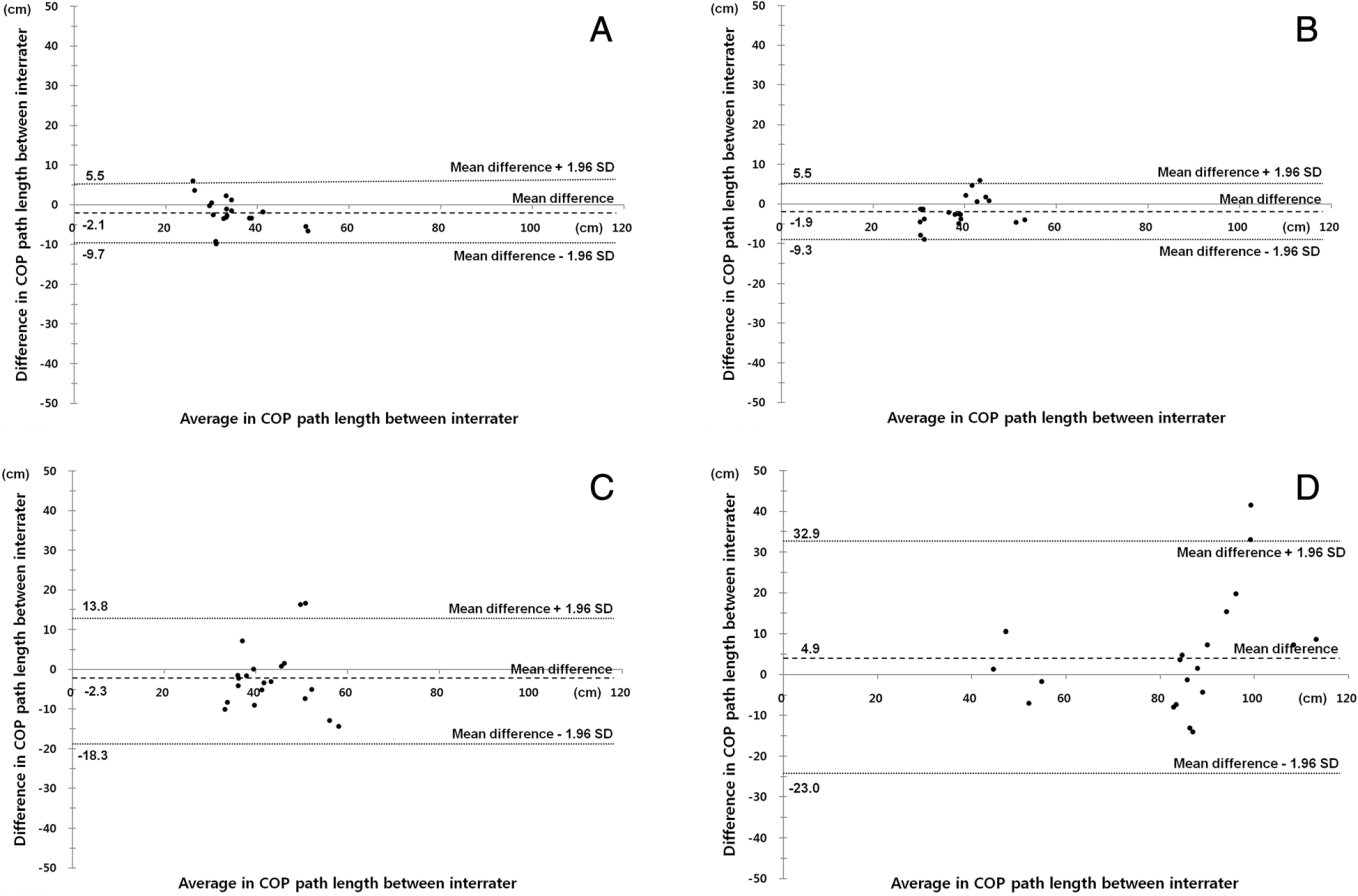
**Bland-Altman plots to assess inter-rater reliability of COP path length.** Inter-rater reliability plots; **(A)** Standing on two legs with open eyes, **(B)** Standing on two legs with closed eyes, **(C)** Standing on one leg with open eyes, and **(D)** Standing on one leg with closed eyes.

Comparison between A1 and B to investigate inter-rater reliability showed in Table 1. The correlation coefficient showed strong correlations for PL and VA of STEO, STEC, and SOEC. The correlation coefficient showed moderate correlations for PL and VA of SOEO. The Inter-rater reliability of the WBB based system appeared highly with all tasks. Also, in Figure 2, the agreement between inter-rater COP path length scores is presented and shows a Bland Altman plot. In this plot, the x-axis represents the mean COP path length of both assessors and the y-axis represents the difference between both measurements. From the Bland-Altman plot, it can be concluded that the agreement between COP path lengths of inter-rater using WBB based system showed ‘good’ reliability.

**Table 1:** Interclass Correlation Coefficients (ICC) and Confidence intervals of postural sway

		**A1**	**B**	**Difference**	**ICC (95% CI)**	**SEM**
STOE	PL(cm)	36.03 ± 7.88	33.92 ± 6.23	-2.11 ± 3.90*	0.918(0.794, 0.968)	1.955
	VA(cm/s)	1.27 ± 0.28	1.19 ± 0.22	-0.08 ± 0.14*	0.918(0.794, 0.968)	0.069
STCE	PL(cm)	39.79 ± 6.37	37.86 ± 7.68	-1.92 ± 3.79*	0.922(0.804, 0.969)	1.899
	VA(cm/s)	1.40 ± 0.22	1.34 ± 0.27	-0.06 ± 0.14	0.915(0.785, 0.966)	0.070
SOOE	PL(cm)	44.42 ± 8.54	42.17 ± 8.57	-2.25 ± 8.18	0.704(0.252, 0.883)	4.090
	VA(cm/s)	4.71 ± 0.94	4.43 ± 0.89	-0.27 ± 0.90	0.685(0.205, 0.875)	0.450
SOCE	PL(cm)	81.03 ± 18.02	85.95 ± 22.70	4.92 ± 14.25	0.862(0.653, 0.946)	7.138
	VA(cm/s)	8.58 ± 1.88	9.10 ± 2.41	0.52 ± 1.47	0.869(0.670, 0.948)	0.735

Numerical data of the scores of tested. The results are given in terms of mean and standard deviation. *p < 0.05 show a significant difference between A1 and B.

**Figure 3:**
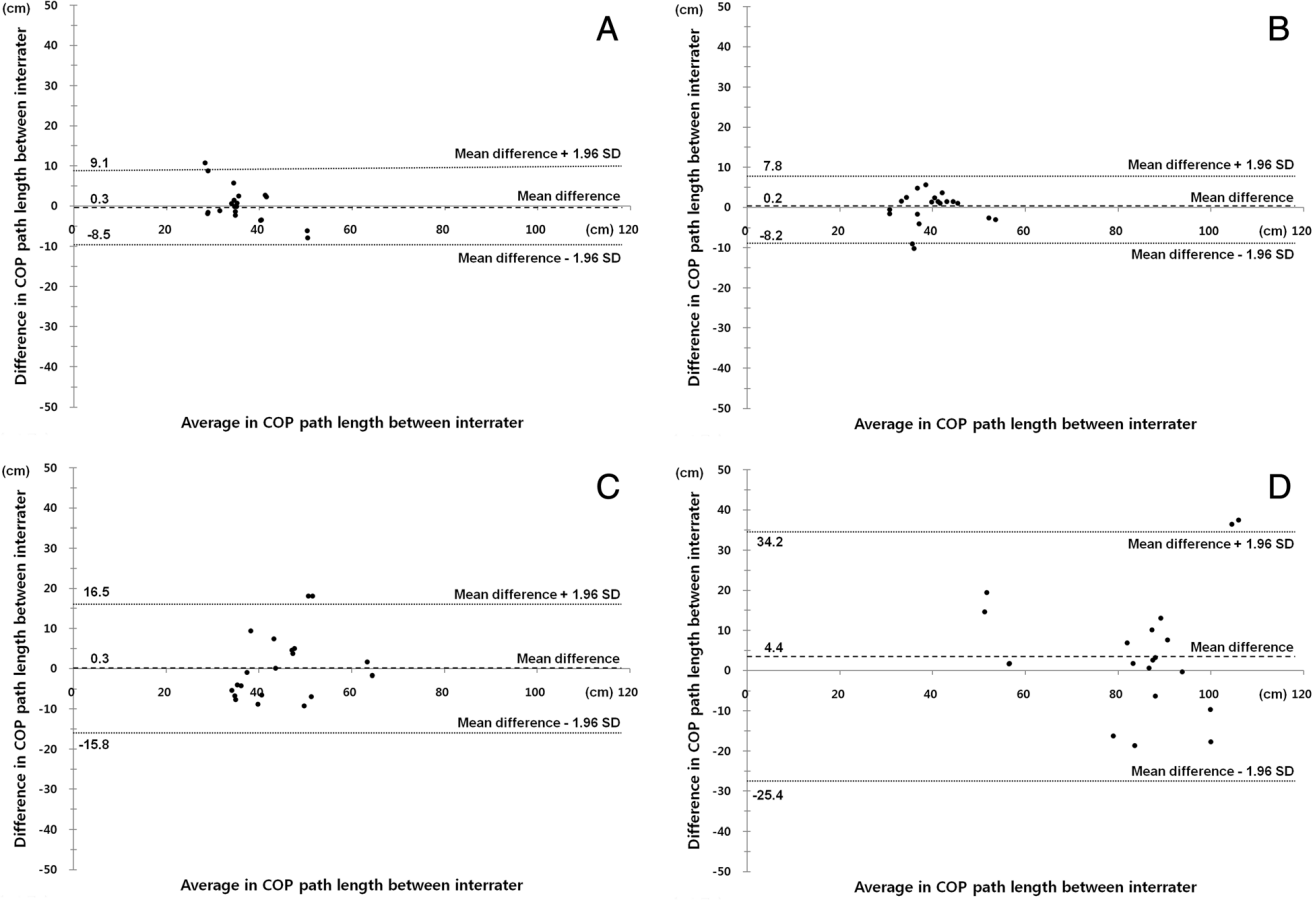
**Bland-Altman plots to assess intra-rater reliability of COP path length.** Intra-rater reliability plots; **(A)** Standing on two legs with open eyes, **(B)** Standing on two legs with closed eyes, **(C)** Standing on one leg with open eyes, and **(D)** Standing on one leg with closed eyes.

Comparison of A1 and A2 to investigate the intra-rater reliability showed in Table 2. The correlation coefficient showed strong correlations for PL and VA of STEO and STEC. The correlation coefficient showed moderate correlations for PL and VA of SOEO and SOEC. The Intra-rater reliability of the WBB based system appeared highly with all tasks. In Figure 3, the agreement between inter-rater COP path length scores is presented and shows a Bland Altman plot.

**Table 2:** Intraclass Correlation Coefficients (ICC) and Confidence Intervals of postural sway

		**A1**	**A2**	**Difference**	**ICC (95% CI)**	**SEM**
STOE	PL (cm)	36.03 ± 7.88	36.34 ± 5.24	0.31 ± 4.50	0.872 (0.678, 0.950)	2.254
	VA (cm/s)	1.27 ± 0.28	1.28 ± 0.19	0.01 ± 0.16	0.864 (0.657, 0.946)	0.082
STCE	PL (cm)	39.79 ± 6.37	39.56 ± 6.54	-0.23 ± 4.08*	0.889 (0.720, 0.956)	2.041
	VA (cm/s)	1.40 ± 0.22	1.39 ± 0.23	0.01 ± 0.14	0.891 (0.724, 0.957)	0.071
SOOE	PL (cm)	44.42 ± 8.54	44.76 ± 10.97	0.34 ± 8.22	0.788 (0.465, 0.916)	4.112
	VA (cm/s)	4.71 ± 0.94	4.73 ± 1.17	0.03 ± 0.90	0.785 (0.457, 0.915)	0.448
SOCE	PL (cm)	81.03 ± 18.02	85.47 ± 18.65	4.44 ± 15.20	0.793 (0.476, 0.918)	7.593
	VA (cm/s)	8.58 ± 1.88	9.04 ± 1.95	0.46 ± 1.61	0.786 (0.458, 0.915)	0.803

Numerical data of the scores of tested. The results are given in terms of mean and standard deviation. *p < 0.05 show a significant difference between A1 and A2.

**Figure 4:**
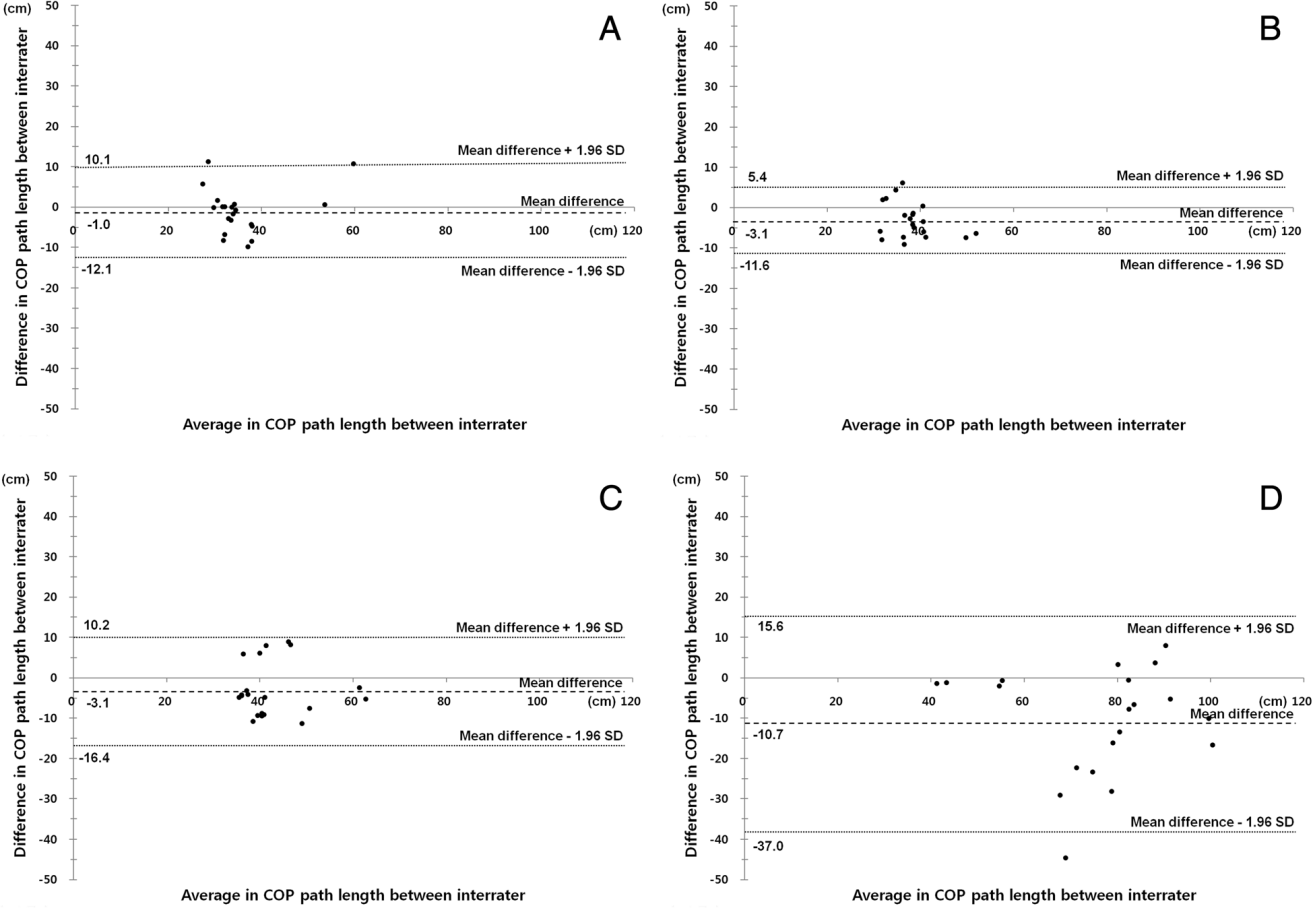
**Bland-Altman plots to assess concurrent validity of COP path length.** Concurrent validity plots; **(A)** Standing on two legs with open eyes, **(B)** Standing on two legs with closed eyes, **(C)** Standing on one leg with open eyes, and **(D)** Standing on one leg with closed eyes.

Comparison between A1 and A3 to investigate the concurrent validity showed in Table 3. The correlation coefficient showed strong correlations for PL and VA of STEO, STEC and SOEO. Although the correlation coefficient showed also strong correlation for PL of SOEC, the correlation coefficient showed moderate correlation for VA of SOEC. It was found that the WBB based system had a high concurrent validity. In Figure 4, the agreement between inter-rater COP path length scores is presented and shows a Bland Altman plot.

**Table 3:** Comparison between WBB based system and laboratory based force platform

		**A1 (WBB)**	**A3 (Force platform)**	**Difference**	**ICC (95% CI)**	**SEM**
STOE	PL (cm)	36.03 ± 7.88	35.03 ± 8.82	-1.00 ± 5.64	0.872 (0.676, 0.949)	5.941
	VA (cm/s)	1.27 ± 0.28	1.17 ± 0.29	-0.10 ± 0.19	0.870 (0.672, 0.949)	0.622
STCE	PL (cm)	39.79 ± 6.37	36.67 ± 4.99	-3.11 ± 4.36	0.831 (0.572, 0.933)	2.175
	VA (cm/s)	1.40 ± 0.22	1.22 ± 0.17	-0.18 ± 0.15*	0.824 (0.556, 0.931)	0.076
SOOE	PL (cm)	44.42 ± 8.54	41.34 ± 8.54	-3.09 ± 6.78	0.813 (0.528, 0.926)	3.390
	VA (cm/s)	4.71 ± 0.94	4.14 ± 0.85	-0.57 ± 0.70*	0.820 (0.544, 0.929)	0.352
SOCE	PL (cm)	81.03 ± 18.02	70.36 ± 17.81	-10.67 ± 13.44*	0.836 (0.586, 0.935)	3.188
	VA (cm/s)	8.58 ± 1.88	6.80 ± 2.01	-1.78 ± 1.79**	0.731 (0.321, 0.894)	0.140

Numerical data of the scores of tested. The results are given in terms of mean and standard deviation. *p < 0.05, **p < 0.01 show a significant difference between A1 and A3.

## Discussion

A reliable and valid balance test device can be advantageous in clinical settings. In particular, such device would be useful for patients with damages of musculoskeletal or nervous systems. In our study, the reliability and validity of WBB-based system for the balance test was investigated. The assessment device was designed to connect the WBB and the laptop through Bluetooth, and the values were analyzed through Balancia software installed on the laptop. Furthermore, this software was designed to analyze variables such as COP path length and COP velocity. The values of these variables can be obtained from a force platform, which are commonly used in laboratories.

The COP path length and COP velocity found from WBB-based system in our study were excellent in the inter-rater reliability and the intra-rater reliability. Concurrent validity was also shown to be consistently excellent across tasks. The results indicate that WBB can be used for measuring balance for patients in clinical settings. Clark et al. [3] reported that WBB as a device for measuring balance while standing is reliable and valid. In particular, high test-retest reliability in terms of the COP path length as well as the high concurrent validity of the WBB was reported. The results of Clark et al. correspond to those obtained in our study. However, in the study by Clark et al., data such as the COP path length was obtained using WBB in conjunction with LabVIEW, while the Balancia design using C++ allowed use with several clicks only. Compared to the method used by Clark et al., the Balancia can be more impressive. Also, in Clark et al’ study, data were sampled at 40 Hz, whereas we transmitted at 50 Hz, which resulted in marginally higher values. In the results for the concurrent validity of our study, a little difference was found in the values of COP path lengths. The differences between the WBB based system and laboratory grade force platform can be attributed to specific factors such as precision and sensitivity of sensor or quality and strength of devices surface. Therefore, the differences prove that there were no affects on the balance test of participants. Accordingly, both a force platform and the WBB can estimate similar values for measuring balance. Thus, our study showed that Wii-based wireless software is a reliable and valid assessment tool.

The Balancia software can be used with WBB; it costs less than US100 and a bluetooth-equipped laptop. There are a lot of advantages of the WBB-based software. We can be less concerned about hardware damage, and we can collect data easily without complicated mathematical analyses using MATLAB. Furthermore, for values such as the COP path length, the WBB-based software can provide a reliable and valid data. Accordingly, the Balancia software is a remarkably inexpensive and efficient device for measuring balance in clinical settings. It can be a bridge for connecting link between the laboratory experiments and clinical settings. However, in our study, the reliability of this device was not investigated for patients with impaired balance caused by damages of the musculoskeletal or nervous system. For patients with impaired balance, such as elderly people or stroke patients, further studies need to be conducted.

## Conclusions

This study investigated the reliability and validity of WBB-based system for the balance test. The results show that the COP path length and COP velocity found from the WBB-based system were excellent in the inter-rater reliability and the intra-rater reliability. Also, concurrent validity was shown to be consistently excellent across tasks. Thus, WBB can be used for measuring balance for patients in clinical settings, and it can become a bridge for connecting the differences between the laboratory experiments and clinical settings.

## Competing interests

The authors declare that they have no competing interests.

## Authors’ contributions

DSP contributed to the design of the study, the interpretation of the results and the software development. GCL contributed to the assessment of patients and the acquisition of data, to draft the manuscript. All the authors read and approved the final manuscript.
